# Context-Specific Genome-Scale Metabolic Modelling and Its Application to the Analysis of COVID-19 Metabolic Signatures

**DOI:** 10.3390/metabo13010126

**Published:** 2023-01-13

**Authors:** Miha Moškon, Tadeja Režen

**Affiliations:** 1 Faculty of Computer and Information Science, University of Ljubljana, 1000 Ljubljana, Slovenia; 2 Centre for Functional Genomics and Bio-Chips, Institute for Biochemistry and Molecular Genetics, Faculty of Medicine, University of Ljubljana, 1000 Ljubljana, Slovenia

**Keywords:** context-specific genome scale metabolic modelling, constraint-based modelling, omics data integration, model extraction method, computational pipeline, metabolic fluxes, context-specific model, COVID-19

## Abstract

Genome-scale metabolic models (GEMs) have found numerous applications in different domains, ranging from biotechnology to systems medicine. Herein, we overview the most popular algorithms for the automated reconstruction of context-specific GEMs using high-throughput experimental data. Moreover, we describe different datasets applied in the process, and protocols that can be used to further automate the model reconstruction and validation. Finally, we describe recent COVID-19 applications of context-specific GEMs, focusing on the analysis of metabolic implications, identification of biomarkers and potential drug targets.

## 1. Introduction

Genome-scale metabolic models (GEMs) have found numerous applications in different domains, ranging from biotechnology to systems medicine [1]. One of their main benefits is that they can provide genotype-to-phenotype projections, such as growth rate and nutrient uptake predictions, and predictions of metabolic flux values. The latter can be used to assess metabolic reaction activities in different contexts [2,3]. A GEM describes a metabolic network with a stoichiometric matrix [4] and each reaction is constrained by its minimal and maximal flux bounds. Moreover, a GEM usually encodes the information on gene–protein reaction (GPR) associations, which can be applied in the adaptation of a GEM to a specific context described with high-throughput data, such as transcriptomics or proteomics data. Such integration can be performed with the application of context-specific model reconstruction algorithms, which are used to adapt the flux bounds of a reference model to a given context described with (at least one) high-throughput dataset. This allows one to at least partially automatise the reconstruction of tissue-specific, cell type-specific, disease-specific, or even personalised GEMs. Further investigation of context-specific GEMs includes comparative analyses between different conditions (e.g., analysis of metabolic reprogramming in cancer cells [5]), and identification of biomarkers and therapeutic targets in different diseases or disorders [6].

Herein, we overview the most popular algorithms for the automated reconstruction of context-specific GEMs using high-throughput experimental data. We also briefly review the different datasets and databases applied in the process. Moreover, we describe different protocols that can be used to further automatise the model reconstruction and its validation. Finally, we describe the state-of-the-art applications of context-specific GEMs on the analysis of metabolic implications of COVID-19, and identification of COVID-19 biomarkers and potential drug targets.

## 2. Genome-Scale Metabolic Modelling

Genome-scale metabolic models (GEMs) aim to systematically encode our knowledge of the metabolism of an organism. Reference GEMs describing generic models of a cell are constructed with a combination of automated approaches and manual curation. Such reconstructions are based on genome annotation data and a myriad of additional data sources, including biochemical databases, organism-specific databases, experimental data, and literature data [7]. GEM reconstruction, its refinement, adaptation, and analysis are commonly performed with the aid of model building tools [8] and reconstruction and analysis frameworks, such as COBRA [9], COBRApy [10], RAVEN [11] or PSAMM [12]. These frameworks provide implementation of a vast scope of methods with different goals, including the reconstruction of a draft metabolic model [13], visualisation of metabolic maps (e.g., see Paint4Net [14]), identification of blocked reactions and gap filling [15] and analysis of reconstructed GEMs, such as optimal steady-state flux assessment [16] or flux sampling [17]. GEMs have been reconstructed for more than 1,000 different organisms [18]. Moreover, advances in our knowledge guide iterative refinements of GEMs. For example, Recon presents a generic human GEM that has gone through several iterations from Recon 1 [19] to Recon 2.2 [20] and to Recond3D [21], and was later extended and integrated with the HMR2.0 database [22] to obtain the Human–GEM model [23].

In the context of biomedicine, GEM applications range from the identification of disease biomarkers to the prediction of drug targets [24], drug repurposing [25] and cancer research [26]. GEMs can also be applied in a vast array of bioengineering applications [18]. These range from predicting cellular phenotypes (e.g., in the context of predicting maximal growth in different conditions and identification of an optimal medium [27]) to guiding metabolic engineering (e.g., in the context of optimal strain design [28]) and identification of a minimal gene set [29].

Most computational approaches aimed at the analysis of GEMs rely on constraint-based modelling and are based on flux balance analysis (FBA) [16] or its derivations. FBA aims to find the metabolic flux values that are consistent with a set of given constraints (minimal and maximal flux bounds) and which bring the system to a steady state. Moreover, FBA requires a specification of required metabolic functionality (RMF) that is used to define an objective function for optimisation. The optimisation can then be formulated as a linear programming (LP) problem. However, since the constraints in this formulation are usually mathematically underdetermined [30], several nonunique optimal solutions exist. To assess metabolic flux ranges through reactions that bring the system to a near optimal, or optimal, steady state, flux variability (FVA) can be used [31]. However, the latter still requires the specification of a RMF, which is hard to identify in a general context and may yield biased results. Moreover, it has been shown that the definition of the RMF strongly affects the precision of model predictions [32]. An unbiased alternative to methods relying on RMF-based optimisation is to use flux sampling of the feasible solution space without a specific optimisation criterion [17].

**Figure 1:**
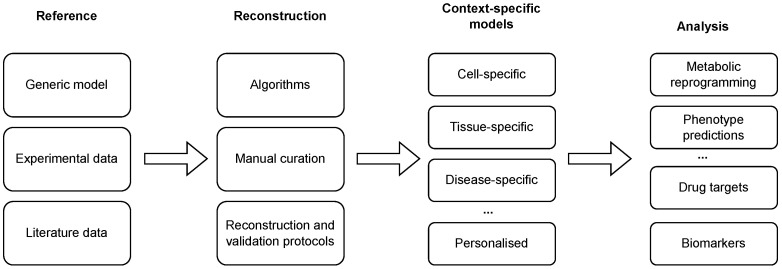
Reconstruction and analysis of context-specific GEMs. Generic models are tailored to a specific context with the integration of (high-throughput) experimental and literature data using a combination of automated algorithms and manual curation. The reconstruction process can be additionally enhanced with the application of reconstruction and validation protocols. The reconstructed models can be used to conduct different analyses, ranging from the prediction of phenotypes and context-specific reprogramming of a metabolic network to the identification of drug targets and disease biomarkers.

Reconstructed GEMs, as described above, present the metabolism of a general cell in an arbitrary context and, thus, compose generic models. Since only specific metabolic reactions are, in fact, active in a specific cell [33], these models need to be further tailored to a specific context in which only a subset of enzymes is active [34]. This process can be carried out using different reconstruction algorithms, in combination with high-throughput datasets and available biological knowledge, to obtain context-specific models (see Figure 1 and Tables 1 and 2). The latter present a subset of a generic GEM and can be used to describe the metabolism of a specific cell in a specific context [35]. Finally, such a model can describe a cell-, a tissue-, a disease-, or even an individual-specific model.

## 3. Algorithms and Tools for Reconstruction of Context-Specific Models

Most algorithms for the reconstruction of context-specific GEMs rely on transcriptomics data to identify active and inactive genes and to adjust metabolic reaction activities in a given context (see Table 2). In this case, each transcript and its corresponding protein/enzyme needs to be associated with specific reactions. One of the first attempts to correlate gene expression data with metabolic flux constraints was presented by Akesson et al. [36]. This was performed on a gene-by-gene basis, where fluxes through the metabolic reactions, with experimental evidence suggesting the absence of their enzymes, were constrained to 0.

Gene–protein reaction (GPR) rules present an association between a specific gene and a metabolic reaction in a model. These rules can describe different types of gene–reaction linkage. For example, different genes might encode different subunits of the same enzyme. In this case, a reaction catalysed by this enzyme can be active only when all of the respective genes are expressed (AND rule). Different genes might also express isoforms of the same enzyme. In this case, a reaction catalysed by this enzyme can be active when at least one of the respective genes is expressed (OR rule) [37]. A large number of recent algorithmic approaches for the reconstruction of context-specific GEMs rely on GPR rules to project the transcriptomics data to reaction activities (see more detailed descriptions of specific algorithms below). However, as illustrated above, GPR rules are encoded in a model as Boolean functions. On the other hand, gene expression data are usually described with non-binary values. In this case, logical OR can be interpreted as the maximum, and logical AND as the minimum, between two or more values (Min/Max GPR mapping) [38]. Alternatively, AND can also be interpreted as the geometric mean, and OR as the sum of two or more values [39].

Certain algorithms only require a definition of a core set of reactions, which are active in a given context. A list of such reactions can be compiled manually (e.g., see [40]) or automatically using transcriptomics data (e.g., see [41,42]). Some reconstruction algorithms allow the integration of other kinds of data, for example metabolomics or proteomics data (see Table 2). A more detailed process of such integration is described, together with a specific reconstruction algorithm, in the continuation of this section.

**Figure 2:**
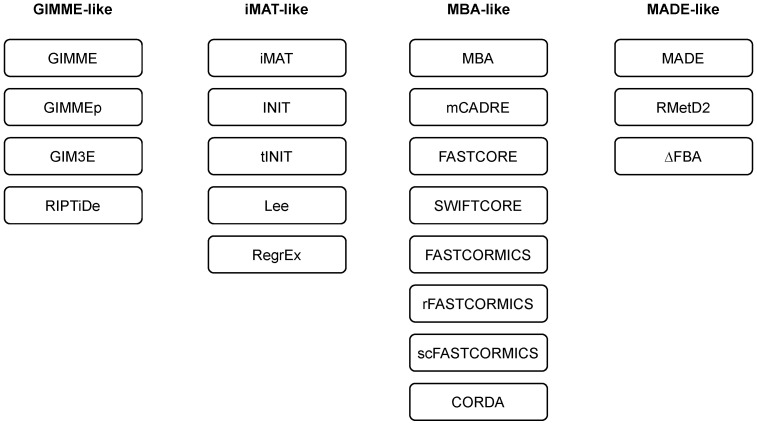
Families of algorithms for automated reconstruction of context-specific models.

We employ and extend the classification of methods as introduced in [43]. Namely, the majority of the methods can be classified into three main families, i.e., GIMME-, iMAT-, and MBA-like families. We also introduce a MADE-like family, which employs differential expression data in the reconstruction process (see Table 1 and Figure 2). An overview of the reviewed algorithms is summarised in Table 2.

### 3.1. GIMME-like Family

The main goal of GIMME-like algorithms is to produce a model that is consistent with the provided experimental data while having the capability to conduct a required metabolic function (RMF). The latter is specified as an additional artificial reaction in the model, usually corresponding to the biomass accumulation [59]. Most of the methods belonging to the GIMME-like family perform reconstruction in the following way: (1) maximisation of an RMF on the basis of FBA and with (2) minimisation of the penalty function describing the inconsistency between the obtained reaction fluxes and the experimental data while maintaining the flux through the RMF above a predefined fraction of the flux calculated in step (1) [43]. GIMME (Gene Inactivation Moderated by Metabolism and Expression) presents the oldest context-specific model reconstruction algorithm to apply gene expression transcriptomics data in combination with the RMF to describe a given context. It generates two irreversible reactions per reversible reaction and solves a linear programming (LP) problem minimising the fluxes through the reactions with corresponding expression levels below a threshold. Here, the penalty is proportional to the difference from the threshold. Only reactions with negative evidence (low expression values) are penalised, while reactions with missing data are omitted from the optimisation process [38].

The extensions of GIMME include GIMMEp [44] and GIM^3^E [45]. GIMMEp allows for the additional integration of proteomic data as an RMF [44]. A separate model is constructed for each proteome-associated reaction objective using the original version of the GIMME algorithm [38]. These models are then combined into a final GIMMEp model. GIM^3^E is another GIMME-based extension that allows the integration of metabolomics data [45]. The turnover metabolite is introduced for each reaction producing or consuming metabolites present in the metabolomics dataset. A sink reaction is added for each turnover metabolite and its lower flux bound is set to a small positive value. This ensures compliance of the network with the metabolomics data, since all detected metabolites need to be used by the network. Similar to the case of GIMME, transcriptomics data are applied to penalise the obtained network. The penalty for a transcript-associated reaction is proportional to its flux and the difference between the maximal gene intensity and the specific gene intensity. The minimal total penalty through the network is then calculated and the optimisation problem is constrained to a value proportional to the minimal total penalty. However, each reversible reaction in a network needs to be converted to two irreversible reactions, and only one of these can be active at the same time. The optimisation problem thus needs to be converted to a mixed integer linear programming (MILP) problem, which increases the computational complexity of the algorithm.

RIPTiDe (Reaction Inclusion by Parsimony and Transcript Distribution) [46] is based on quantitative transcriptomic data and aims to find the most cost-effective use of metabolism in a way similar to RegrEx, which can be classified into the iMAT-like family (see Section 3.2 and Reference [51]). However, RIPTiDE requires the specification of a RMF and is based on a formulation similar to parsimonious enzyme usage FBA (pFBA) [60] to minimise overall flux values through the network [46]. RIPTiDe additionally weights each reaction on the basis of quantitative expression data. Firstly, it maximises the flux through the RMF to evaluate the optimal solution of the generic model, and then constrains the next optimisation steps to a near optimal solution value in a similar manner as GIMME [38]. Based on the gene expression profiles, a linear coefficient (weight) from the interval (0,1] is evaluated for each reaction, whereas higher transcript abundances correspond to lower coefficient values. The median coefficient value is assigned to reactions that are not described in the experimental data. RIPTiDE then minimises the weighted sum of flux values pertaining to at least the minimal required flux through the RMF. Finally, reactions and pathways with zero flux values are removed from the network. RIPTiDE can also be used to analyse the extracted network by observing the inverse linear coefficients of each reaction, and by flux sampling on the constrained and reduced model [46].

GIMME-like algorithms have been widely applied in the past. For example, GIMME and its extensions have been used for the reconstruction of context-specific *Escherichia coli* and human cell models [38], for the extraction of cancer-specific GEMs [61], in the analysis of metabolic immunomodulators of macrophage activation [44], and in the analysis of *Salmonella Typhimurium* metabolism in different media [45]. Applications of RIPTiDE include the prediction of metabolic patterns of *Escherichia coli* [46] and the reconstruction of *Clostridioides difficile* cells in infection and in in vitro settings [62].

One of the main limitations of the GIMME-like methods is that they focus on the optimisation of RMF, which might lead to inconsistencies between reaction fluxes and experimental data [63]. Another drawback of the GIMME-like methods is that they require the definition of the RMF, which is hard to define in a general setting [64,65] as the general applicability of biomass accumulation is questionable [50,66]. If the RMF is unknown for a given context, alternative reconstruction algorithms should be employed. These are described in Section 3.2 and Section 3.3.

### 3.2. iMAT-like Family

In contrast to the GIMME-like family, the family derived from iMAT does not require an exact definition of the RMF. The reconstructed context-specific network presents a solution of MILP classifying reactions in a reference model as active or inactive to comply with the corresponding states of experimental data [43]. This means that quantitative experimental data need to be classified into two or more groups describing different states of data (e.g., expressed and not expressed in the context of transcriptomics data).

The integrative Metabolic Analysis Tool (iMAT) [47] presents an implementation of the method previously proposed by Shlomi et al. [67]. It allows the integration of transcriptomic and proteomic data in which each gene or protein is described with one of three states, namely low, moderate or high expression. The iMAT also performs a discretisation of these data, if necessary, and predicts the flux activity state of each reaction (active or inactive), based on the maximisation of matches between the reaction state and the corresponding transcript/protein state obtained from experimental data. This can be performed by solving an MILP problem that has many alternative solutions. A variant of flux variability analysis (FVA) [31] is employed to account for these solutions. For each reaction, maximal attainable similarity with the expression data is calculated for a condition in which the reaction is always active and for a condition in which the reaction is always inactive. The reaction is deemed active if its inclusion increases the similarity with the experimental data and is considered inactive if its inclusion decreases the similarity with the experimental data [67].

INIT (Integrative Network Inference for Tissues) was initially developed to employ protein abundance data from the Human Protein Atlas as the main source of data [48]. However, gene expression data could also be integrated when proteomic evidence is not available. Experimental data are used to evaluate the weight of each reaction, where the weights represent arbitrary (discrete) functions of experimental evidence. Each reaction can have a positive (presence) or a negative weight (absence). During MILP-based optimisation, reactions are included/excluded from the model to maximise the sum of weights of included reactions. Additionally, INIT does not presume a steady state of the network, since it imposes a positive net production of metabolites present in the experimental data. Namely, the net production of metabolites with experimental evidence is forced to exceed a given lower bound. Therefore, INIT allows for the qualitative integration of metabolomics data [48].

Task-driven INIT (tINIT) presents an INIT extension that focuses on functional context-specific models, since the models obtained need to perform a given set of metabolic tasks [49]. These might include the production or consumption of a certain metabolite or the activation of a pathway known to be active in a given context. Furthermore, tINIT additionally constrains the reversible reactions so they cannot have flux in both directions simultaneously. The user can specify whether the net production of metabolites is allowed or if the steady-state is imposed. Recently, another variation, i.e., rank-based tINIT, which employs a rank-based weight function, was proposed [61,68].

The approach presented by Lee et al. maximises the correlation between measured gene expression data and predicted reaction fluxes within the model [50]. The approach is similar to iMAT; however, it relies on absolute (continuous) RNA-seq gene expression data. In contrast to other iMAT-like approaches, transcriptomics data are not discretised, and the minimisation of the distance between absolute expression values and reaction fluxes is used during the optimisation. However, the optimisation function is additionally linearised to convert the problem into a computationally more feasible alternative, i.e., a convex programming problem. Model reconstruction is performed through an iterative process involving the following steps: (1) maximise the correlations between irreversible reactions and experimental data using the above-described optimisation function, and (2) use FVA to identify the reversible reactions that reduce the correlation values found in step (1). In step (2) the set of irreversible reactions is increased and steps (1) and (2) are repeated until no additional irreversible reactions are found, or until a predefined number of iterations is performed.

RegrEx extends the approach presented by Lee et al. with the inclusion of regularisation into the optimisation function to enhance the exclusion of reactions that are irrelevant to a context [51]. Moreover, in comparison to Lee’s approach, RegrEx does not require iterative removal of reversible reactions and is unbiased regarding the order in which reversible reactions are removed. Since the Euclidean distance between the experimental evidence and flux values is used during the optimisation, the latter presents a quadratic programme. Finally, RegrEx formulates the optimisation problem as a mixed integer quadratic programme (MIQP) to constrain the activity of a reversible reaction to a single direction only [51].

Applications of iMAT-like methods include the reconstruction of human cell type-specific and cancer-specific GEMS [22,48,61], identification of anticancer drugs [49], predicting of human tissue-specific metabolism [67], and the reconstruction of cell specific models of *Arabidopsis thaliana* [69]. The main limitations of this family derive from the lack of an objective function, which might result in the reconstruction of a nonfunctional model [63]. However, this problem might be addressed with the employment of a set of required metabolic tasks, as introduced in tINIT [49].

### 3.3. MBA-like Family

The MBA-like family is based on the identification of core reactions, which is followed by the removal of the reactions that are not in a core set [43]. Similar to the iMAT-like family, these algorithms do not presume a specific metabolic objective, which makes them applicable to cases wherein the latter is unknown. The first member of the MBA-like family, called the Model Building Algorithm (MBA) [52], employs a set of core reactions that can be identified using multiple data sources and curated biochemical knowledge. Moreover, these reactions need to be further separated into two groups, namely, high- and moderate-likelihood reactions. The goal of the algorithm is to obtain a reconstruction including all high-likelihood reactions, a maximal number of moderate-likelihood reactions and a minimal set of remaining reactions required to fill the gaps in the reconstructed model. The algorithm iteratively prunes randomly selected non-high-likelihood reactions while maintaining the model’s consistency. Since the sequential order of reaction removal affects the obtained results, the procedure is repeated several times to yield a set of candidate models. The final model is obtained on the basis of consensus among these candidate models. Checking of the model consistency might be performed with the application of FVA [31] to assess if the removal of a reaction causes gaps (blocked reaction) in the model. A computationally more feasible alternative identifies a list of reactions that cannot be activated, due to the removal of a reaction by repeatedly conducting the following steps: (1) maximise the fluxes through the reactions in the list; (2) minimise the fluxes through the reactions in the list; (3) minimise/maximise the flux through each reaction in the list. When a non-zero flux is found for a reaction in any of these steps, the reaction is removed from the list.

The mCADRE (metabolic Context-specificity Assessed by Deterministic Reaction Evaluation) applies different types of reaction scores, based on gene expression data and network topology. These scores are applied to identify the set of core reactions, as well as to define the order in which non-core reactions are removed [53]. Reactions with expression scores above a threshold are selected as core reactions. Non-core reactions are ranked according to their connectivity- and confidence-level scores. In a similar way to MBA, the consistency of the model is evaluated after a selected reaction is removed. However, mCADRE allows the user to also define a set of key metabolites that appear in a given context. Furthermore, mCADRE does not require all core reactions to be kept in the final model. Namely, a core reaction can be removed if it does not prevent the production of a key metabolite and if this does not block other core reactions. Non-core reactions can, thus, be omitted in the case of strong experimental evidence of their absence in the context (negative set of reactions) even if they block some of the core reactions. However, the ratio between the number of blocked core reactions and the number of blocked non-core reactions needs to be below a predefined threshold. Consistency checking is performed in a similar way as for MBA, but with the application of the FastFVA algorithm [70].

FASTCORE aims to find a minimal consistent network in which all core reactions, supported by experimental evidence, are active [40]. It identifies a minimal set of sparse modes (i.e., feasible flux vectors) in which all core reactions are active. This is achieved with an iterative application of two linear programmes to maximise the number of reactions with non-zero flux values in the core set and to minimise this number outside the core set [40]. The algorithm exhibits decreased computing time in comparison to mCADRE and MBA, due to the application of the fast consistency checking (FASTCC) algorithm. The latter aims to maximise the function that pushes all the fluxes in the network away from zero. In this way, FASTCC can detect all blocked reactions in a single LP iteration. However, when dealing with reversible reactions, additional LP iteration is required in which reversible reactions are considered for negative flux. To avoid the manual compilation of different datasets for the identification of core reactions, FASCTORE was recently extended to allow direct integration of high-throughput transcriptomics data. While FASTCORMICS [41] presents a pipeline for the direct integration of microarray data, rFASTCORMICS [42] presents another FASCTORE adaptation for the direct integration of RNA-seq data (rFASTCORMICS), and scFASTCORMICS for the integration of single-cell RNA-seq (scRNA-seq) data [55].

SWIFTCORE tackles the reconstruction problem in a similar way as FASTCORE does and even exceeds its performance in the context of reconstruction runtime and network compactness [54]. The algorithm is also based on linear programming. However, increased performance of SWIFTCORE can be attributed mainly to its handling of reversible reactions. It does not determine the direction of a reversible reaction, but encourages one of the possible directions in a soft manner. This approach neither requires a MILP formulation, nor the solving of two LP problems for reversible reactions [54].

Pruning of all non-core reactions leads to a so-called parsimonious reconstruction yielding a minimal model for a given context. However, such an approach might cause the removal of fundamental reactions, for which experimental data are not available [34]. The cost-optimization reaction dependency assessment (CORDA) algorithm aims to solve this problem by loosening the constraint to remove all non-core reactions and, thus, obtain a concise, but not minimal (and unrealistic), reconstruction. [34]. It is based on the assessment of the dependency of reactions with strong experimental evidence on the reactions with little or no experimental evidence. Reactions from the latter two groups are added to the reconstruction if they are associated with a reaction from the first group. CORDA exhibits fast execution times, since it only relies on FBA and solving LPs. Moreover, in contrast to reaction-pruning algorithms, such as MBA and mCADRE, the model reconstruction is independent of the ordering of reactions [34]. It also supports the integration of required metabolic tasks, similar to the tINIT algorithm [49].

Selected applications of MBA-like methods include analysis of cell-type specific epigenetic control points of the macrophage metabolic network [41], analysis of metabolic rewiring in different cancer cells [42], reconstruction of tissue-specific human models [34], and reconstruction of head and neck squamous cells in healthy and cancer states [71].

One of the main limitations of MBA-like methods is that they require a set of core reactions, which need to be compiled manually, while relying on the literature data, biochemical databases, and experimental data. This problem can be at least partially avoided using the FASTCORE extensions that allow direct integration of transcriptomics data [41,42,55].

### 3.4. MADE-like Family

The last group of reconstruction algorithms relies on differential expression data to reconstruct genome-scale metabolic models that describe differences in metabolic fluxes between two contexts/conditions. MADE (Metabolic Adjustment by Differential Expression) uses differential expression data between two or more conditions. For each of the genes observed, these data describe a type of change between the conditions (decrease, increase, or unchanged) and the significance of the change. MADE aims to find a sequence of binary expression states that describe the binarised activity of the genes (i.e., on or off) that most closely match the corresponding differences in expression levels, whereas the statistical significance of these differences is used to create the most probable sequence. This sequence is then used to reconstruct GEMs and obtain metabolic fluxes that describe a specific condition, while maintaining the minimum flux value required through the RMF [56]. The original implementation of MADE was extended further within the TIGER framework (Toolbox for Integrating Genome-scale Metabolism, Expression, and Regulation) to allow multilevel gene expression states and to allow comparison between arbitrary conditions in a sequence [72].

Relative Metabolic Differences version 2 (RMetD2) presents a similar approach, focusing on the integration of differential expression data that describe the difference between two biological conditions [57]. RMetD2 pushes the flux constraints in the direction of experimental evidence. This push is performed in several steps, which allows the evaluation of the consistency of flux changes using standard measures, such as the Spearman correlation between flux values. Contrary to MADE, RMetD2 can operate without the specification of the RMF and can also incorporate additional constraints that describe the perturbed model.

A more recent approach, called ΔFBA, maximises consistency and minimises inconsistency between flux changes and gene expression changes among two conditions [58]. A set of up- and down-regulated reactions is obtained from the differential expression data using Min/Max GPR mapping. These are then integrated into a MILP optimisation in which flux differences between the conditions are constrained to a steady state. Flux differences can be additionally constrained on the basis of experimental data. MILP is then used to maximise the consistency or reaction fluxes with gene expression changes. The algorithm additionally aims to find a minimal solution with the minimisation of the L2 norm of flux differences between the conditions. Similar to RMetD2, ΔFBA does not require the specification of the RMF.

## 4. Data for Model Construction and Validation

As described in the preceding sections different types of high-throughput data can be used in combination with context-specific model reconstruction algorithms (see Table 3), these being transcriptome, proteome and metabolome data. The majority of reconstruction algorithms use the gene or protein expression value, or the metabolite concentration, as input data (see Section 3). Alternatively, algorithms including MADE-like family [56,57,58] and METRADE [73,74] can use the differential gene or protein expression level as input data.

The datasets used in the reconstruction of GEMs can be found in open-access repositories. The open-access repositories impose community-developed reporting standards, which include a set of minimum information data and meta-data being required and validated at the deposition of the data. This is true for the repositories at NCBI and EBI, which govern the majority of repositories. The omics data not deposited in data repositories have less meta-data associated with it and is available in a much less standardised form. However, even data in standardised repositories have a problem regarding missing information, poor experimental design and mis-annotation of samples.

The transcriptome encompasses all RNA molecules expressed by an organism. Transcriptomics are methods used to analyse transcriptomes. There are two major methods, DNA-microarrays and RNA sequencing by NGS (next-generation sequencing). Both technologies generate data describing gene expression value or differential gene expression value for each gene. Such data is usually deposited in public repositories and is, therefore, easily accessible. There are general repositories at NCBI (National Center for Biotechnology Information) and EBI (European Bioinformatics Institute), where researchers can upload any transcriptomic data from humans and plants to bacteria. COVID-19-related transcriptomic datasets can be retrieved from these repositories, namely, GEO (Genome Expression Omnibus) [75,76] (https://www.ncbi.nlm.nih.gov/gds, accessed on 1 November 2022), Array Express [77] (https://www.ebi.ac.uk/arrayexpress, accessed on 1 November 2022), SRA (Sequence Read Archive) (https://www.ncbi.nlm.nih.gov/sra, accessed on 1 November 2022), Expression Atlas [78] (https://www.ebi.ac.uk/gxa/, accessed on 1 November 2022), and ENA (European Nucleotide Archive) (https://www.ebi.ac.uk/ena, accessed on 1 November 2022).

There are many dedicated public data repositories available, which collect other data besides that of transcriptomes and enable a comprehensive synthesis of information. The Genotype-Tissue Expression (GTEx) database (https://gtexportal.org, accessed on 1 November 2022) is a resource dedicated to the study of tissue-specific gene expression and regulation in non-diseased human tissue and single cells. The European Genome–Phenome Archive (EGA) [79] (https://ega-archive.org/, accessed on 1 November 2022) is an archive of genomic, transcriptomic, phenotypic and clinical data from medical research projects. FANTOM5 (Functional Annotation of the Mammalian Genome 5) is another resource with transcriptomic data, which also includes non-coding RNA, in combination with information about the regulatory elements in mammalian cells [80] (https://fantom.gsc.riken.jp/, accessed on 1 November 2022).

Many repositories are disease-oriented, collecting and combining not only transcriptomic data, but also proteomic data. Many of these are dedicated to cancer. The main one is The Cancer Genome Atlas (TCGA) [81]. This is a repository for genomic, epigenomic, transcriptomic, and proteomic data from over 30 different cancers (https://www.cancer.gov/tcga, accessed on 1 November 2022). The omics data is complemented with clinical and imaging data, enabling a classification of samples in analyses. The cBio Cancer Genomics Portal is also dedicated to integrating multidimensional cancer-related omics datasets [82] (https://www.cbioportal.org/, accessed on 1 November 2022). The Cancer Cell Line Encyclopedia (CCLE) is a repository of transcriptomic and proteomic datasets from cancer cell lines [83] (https://sites.broadinstitute.org/ccle/, accessed on 1 November 2022).

The proteome encompasses all the proteins expressed by an organism. Proteomics are methods by which we analyse the proteome, these being two-dimensional gel electrophoresis, different types of protein-microarrays and mass spectrometry. Using proteomic methods, we can measure protein gene expression level or differential protein expression. The ProteomeXchange is an international consortium dedicated to the standardised archiving and dissemination of mass spectrometry-based proteomic datasets and tools for their visualisation and analyses [84] (http://www.proteomexchange.org/, accessed on 1 November 2022). The Human Protein Atlas (HPA) includes data on gene and protein expression collected from different omics experiments [33] (https://www.proteinatlas.org/, accessed on 1 November 2022). The National Cancer Institute’s Proteomic Data Commons (PDC) is a proteomics repository dedicated to cancer-related data and enables integration with genomic and medical image datasets (https://pdc.cancer.gov/pdc/, accessed on 1 November 2022).

The metabolome represents all metabolites that are present in an organism. Metabolomic methods are based on the chromatographic separation of metabolites, coupled with mass-spectrometry, which enables the identification and quantification of the metabolome. Currently, there are a few metabolomic repositories available, such as the Metabolomics Workbench (https://www.metabolomicsworkbench.org/, accessed on 1 November 2022) and the MetaboLights [85] (https://www.ebi.ac.uk/metabolights/studies, accessed on 1 November 2022).

## 5. Reconstruction and Validation Protocols

**Figure 3:**
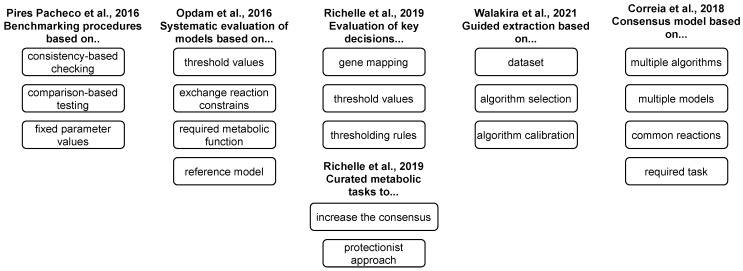
An overview of selected benchmarks and protocols to guide the extractions of context-specific models [35,86,87,88,91,93].

Although specific reconstruction algorithms aim towards full automation (e.g., see ΔFBA [58]), most of the algorithms require evaluation of certain parameters (such as gene activity thresholds). Moreover, different algorithms employ different presumptions during the reconstruction (see Section 3), and they might produce significantly different results for the same dataset. It has been demonstrated that model content might reflect larger variation, due to the employed algorithm more than to the cell type [86]. Thus, it is vital to select the algorithm that yields the reconstruction with the highest biological significance in a given context. Specific benchmarks and protocols have been developed to guide the selection of the most appropriate model extraction method, as well as its calibration, and to increase the quality of reconstructed models (see Figure 3).

Pires Pacheco et al. presented an overview of benchmarks for testing different context-specific model reconstruction algorithms [87]. They divided the methods into the following two groups: Consistency-based testing can be used to analyse robustness against missing or noisy data, as well as capability of methods to distinguish among different contexts. It includes approaches such as cross-validation, adding noise to expression data, and estimating the diversity of generated models from similar and diverse cell groups. The second group is that of comparison-based testing, which is based on comparing the functionalities with other models, manually curated networks or additional datasets. On the basis of these tests, the authors proposed a benchmark for testing context-specific reconstruction algorithms on real and artificial input data. While the models built upon real data were tested for required metabolic functionalities, the artificial data models were built on different fractions of artificial data and the output models were compared to the complete input model. The obtained results indicate that the models constructed with different algorithms vary considerably, even when constructed using the same data. The performed tests indicate that the algorithms that performed discretisation of gene expression data yield better results in the context of inclusion and activity of reactions, supported by experimental evidence; and express better predictive power. Algorithms that consider unknown data as absent (exclusive methods) showed better predictive power than generic models. On the contrary, algorithms that considered unknown data as present (inclusive) tend to generate larger networks, and scored lower when comparing the networks to new data, but were more robust to noisy data and had reduced resolution power. This was evident from the cross-validation experiments. GIMME-like algorithms performed the best in the context of noise robustness. However, algorithms from the MBA-like family (i.e., FASTCORE and FASTCORMICS) performed the best in capturing the variability between different tissues. An important aspect to consider was also the computing speed, which could range from seconds (e.g., FASTCORE) to hours (e.g., GIMME and INIT).

Pires Pacheco et al. [87] aimed at keeping the parameters used for reconstruction in accordance with the values used in the original implementations. However, Opdam et al. systematically evaluated different cancer cell line models obtained by using six different reconstruction algorithms (i.e., FASTCORE, GIMME, iMAT, INIT, MBA, and mCADRE) in combination with different gene expression thresholds, three sets of uptake and secretion flux constraints, and with slightly modified RMF, or without the definition of the RMF [35]. Moreover, Recon 1 [19] and Recon 2.2 [20] were used as template models [19,20]. In the experiment, they applied RNA-seq data from cancer cell lines. They analysed the reactions that were identified to be active in each of the models and tested their predictive capabilities on gene-essentiality predictions and on the metabolic functionalities of the obtained models. The selection of a template model and composition of the RMF did not significantly affect the qualitative response of the reconstructed models. The authors analysed the effects of other factors with the application of principal component analysis (PCA) on the set of reactions present in each context-specific model. While gene expression threshold had the strongest effect (largest amount of explained overall variance in the first principal component), the selection of the reconstruction algorithm had a moderate effect and flux constraints only had a significant effect on the third principal component. Furthermore, the authors compared the accuracy of gene-essentiality predictions across the obtained models. All context-specific models had higher accuracies compared to the generic GEM. However, more stringent threshold cutoffs resulted in more accurate gene-essentiality predictions, and different algorithms yielded different accuracy levels. The most accurate predictions were obtained using INIT, MBA, and mCADRE algorithms with genes above the top 10% of the gene expression level as active, and genes below the mean expression level as inactive. Even though expression thresholds had the largest impact on the reactions included in a model, algorithm selection had the largest impact on model accuracy. Furthermore, the authors tested whether 56 metabolic functions required for cancer growth, encoded as a biomass function, were maintained in the extracted models. The models were constructed without specifying this as a RMF, except in the case of the GIMME algorithm. They evaluated the functionality score by comparing the number of functionalities that a model was able to perform with the number of functionalities performed by models constructed from randomised data. PCA-based analysis indicated that gene expression thresholds had the strongest effect on functionality scores and algorithm selection had a moderate effect. Some functionalities were omitted from most models, which could also be attributed to missing or incomplete GPR associations encoded in a generic GEM. Richelle et al. presented a framework to address this problem with a list of curated metabolic tasks that a cell should perform and that were protected during model reconstruction [86]. Their approach increased the consensus among models reconstructed with different extraction algorithms on 44 cancer cell lines. In addition, the extracted models better captured the biological variability between the cell lines. For each cell line, the authors inferred active metabolic tasks from the list of curated tasks using transcriptomic data and integrated these into an MBA-like family of methods. The same approach could not be applied to IMAT- and GIMME-like methods, in which the protectionist approach would require a modified implementation.

In another article, Richelle et al. focused on the evaluation of key decisions that must be made in the integration process of transcriptomic data [88]. They focused on the initial preprocessing steps of data integration, namely on the possible interpretations of GPR associations (gene mapping) and the selection of gene expression thresholds to identify active and inactive genes (thresholding). The authors used two different gene mapping approaches, namely the Min/Max and Min/Sum GPR mapping. While in the first, Boolean OR was interpreted as maximum, in the second it was interpreted as a sum. Furthermore, the authors proposed three different thresholding rules. The first option was to have a single threshold in a global context (global T1). A gene-specific threshold could be evaluated when multiple samples were available (local threshold). This could be combined with a global concept for a set of genes with low expression values across all the samples to prevent their inclusion in a set of active genes (local T1). The third rule (local T2) applied the local rule only to genes having expression between predefined lower and upper bounds to also address anomalies that might arise within the family of genes with high expression levels in all samples. Not only was the selection of gene mapping method applied important in combination with a thresholding rule, but also the order in which these two steps were applied was important. To assess the impact of these decisions, the authors used different combinations of preprocessing steps on the data describing 32 tissues to obtain 640 reaction lists for evaluation. They showed that the thresholding rule had the largest effect on variability in the obtained list of reactions. Interestingly, this had an even larger effect than differences among tissues. The order of preprocessing had a significantly smaller effect, and gene mapping had the smallest influence among all observed factors. The local T2 rule, with thresholding of gene expression before conversion to reaction activity, yielded the most accurate results in the context of tissue grouping at the reaction level. The local T2 rule also reduced the number of false negative predictions at the tissue level and provided the most accurate list of active reactions. The result obtained by a T2 rule was later additionally improved using a heuristic method, StanDep, that firstly clusters the data, based on their gene expression patterns in different contexts, and, then, determines the thresholds within each cluster separately [89].

One still needs to select the most suitable extraction methods for a given context. According to the no-free-lunch theorem, an optimisation method that would supersede all other methods in all cases does not exist [90]. This also holds for the algorithms for context-specific GEM reconstruction. A specific method must be selected and calibrated together with a given dataset to yield the best results. Walakira et al. [91] proposed a protocol to guide the selection of the most suitable algorithm and its configuration in dependence on the applied data. This selection was, on the one hand, guided by the heterogeneity of the extracted models and, on the other hand, by the ability to capture the true variability in the provided data. The latter was based on the evaluation of the explained variability by the observed factors within each algorithm. These factors might include cell type, genotype, gender of an organism, or diet used in the experiment. The authors demonstrated their approach in the evaluation of five different extraction algorithms (GIMME, iMAT, FASTCORE, INIT and tINIT) in a combination with different thresholding values and threshold rules (see Ref. [88]) in an analysis of *Cyp51* knockout mice diet experimental data [92]. They showed that, in their specific case, the models with the largest biological relevance were extracted using the FASCTORE algorithm and using the 80th percentile of gene expression values between all genes in a sample as a gene expression threshold value to determine the core set of reactions.

An alternative approach does not focus on the selection of the best single approach for a given dataset. Instead, the objective is to address the issue of algorithm heterogeneity by constructing a consensus model from the set of models constructed using different extraction algorithms [93]. The proposed method builds a consensus model from the reactions present in most of the models, and then iteratively extends this model with the additional reactions required to perform a predefined set of metabolic tasks. The authors demonstrated that the obtained consensus model yielded more accurate results in the context of the prediction of known metabolic phenotypes and traits in the experimental data [93].

## 6. COVID-19 Applications of Context-Specific Genome-Scale Metabolic Modelling

The COVID-19 pandemic has significantly impacted our personal and work lives. The disease is caused by infection with SARS-CoV-2, a virus from the Coronaviridae family, causing severe acute respiratory syndrome, and has resulted in millions of deaths worldwide. In combatting COVID-19 all available resources in science have been harnessed, not only experimental tools, but also computational tools. There were several omics datasets generated by analysing SARS-CoV-2-infected human samples and various cell lines, which are available in GEO, ENA, EGA and ArrayExpress repositories. Numerous papers have been published reporting on experiments studying viral biology, virus effects on cell metabolism, immune cells and the overall effect on the immune system and the human body.

**Figure 4:**
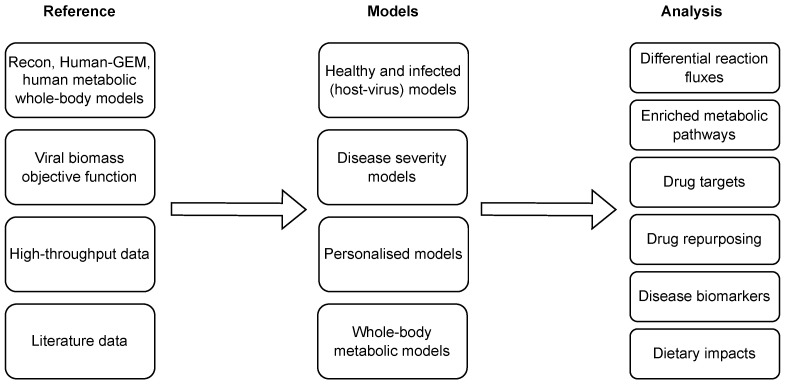
Reconstruction and analysis of COVID-19-specific GEMs. Recon models, Human–GEM and human metabolic whole-body models (WBMs) have been applied as reference models in combination with different forms of viral biomass objective function (VBOF), high-throughput data, and literature data. The reconstruction approaches have focused on establishing group-specific, or even personalised healthy and infected (host-virus) models with different disease severity levels, and different complexities (tissue-specific or whole-body models). The reconstructed models have been used to conduct differential flux and pathway enrichment analyses, to identify possible drug targets, disease biomarkers, and dietary impacts on COVID-19 metabolic reprogramming.

Several computational approaches have been applied to combat COVID-19, [94] ranging from the establishment of a knowledge repository of COVID-19 molecular mechanisms, i.e., COVID-19 disease maps [95,96], to the identification of candidate drugs which may be helpful in COVID-19 treatment and prevention [97]. In this context, different approaches have also been proposed to analyse the COVID-19 metabolic signatures and propose novel treatments and diagnostic approaches using GEMs. Figure 4 presents the state-of-the-art COVID-19-related applications of context-specific GEMs, which are additionally overviewed in Table 4, and described in more detail in this section.

Renz et al. generated a host–virus model of human alveolar macrophage [98], based on an approach that had been previously applied to other viruses [111]. The reconstruction of a host–virus model was based on a manual integration of a SARS-CoV-2 biomass objective function (VBOF) into the host model, i.e., iAB-AMØ-1410 model [112]. The VBOF was reconstructed on the basis of available knowledge considering amino acids, nucleotides, and energy requirements of the virus. The obtained model was then used to analyse the stoichiometric changes between host and virus by comparing host and viral biomass objective functions (BOFs), and metabolic changes between host- and virus-optimised states using FBA and FVA. The latter was used in the identification of potential antiviral targets. The obtained results suggested that supplementation of L-isoleucine and L-lysine, and the inhibition of guanylate kinase, ought to be the targets. The latter was confirmed by a follow-up study using a refined model, in which different SARS-CoV-2 variants were also considered [99].

Delattre et al. performed a similar analysis using a human lung cell model [100]. The latter was adapted from the Recon 2.2 model [20] on the basis of gene expression data from the Human Protein Atlas [33]. The obtained model was then extended with the VBOF constructed on the available literature data in a similar way to that described in [98,111]. Simulations of the obtained model were conducted using the FBA. By using the VBOF as an objective, the authors proposed a set of individual and double perturbations as potential drug targets to inhibit SARS-CoV-2 reproduction in a host cell. In addition, they identified a set of existing drugs that complied with the proposed targets.

The VBOFs proposed by Renz et al. [98,99] and Delattre et al. [100] were later extended by Wang et al. to increase their accuracy and to account for the Alpha and Delta variants of COVID-19 [105]. These VBOFs were integrated into Recon3D [21] to obtain variant-specific models. These were then applied to identify antiviral enzymes and metabolites using a fuzzy hierarchical optimisation framework, in which identified antiviral targets presented the smallest possible metabolic perturbations, eliminated virus replication, and allowed the infected cells to restore the dynamics of healthy cells. The optimisation problem was applied to investigate reactions modulated in both gene-centric (identification of reactions regulated by enzymes) and metabolite-centric (identification of reactions related to a metabolite). It revealed dihydroorotate dehydrogenase inhibitors and different two-target combinations to block viral biomass growth. They also identified a set of drugs from the DrugBank database [113] as potential candidates for COVID-19 drug repurposing. Furthermore, according to the metabolite-centric approach, inhibition of CTP and UDP revealed a similar effect to molnupiravir, which reduced the risk of hospitalization or death in at-risk, unvaccinated adults with COVID-19 [114].

Santos-Beneit et al., 2021 [101] reconstructed context-specific models of healthy lung tissue using pyTARG [115], a variant of the PRIME algorithm [116]. Since these algorithms require an association of transcriptomic data with phenotypic measurements (in a manner similar to METRADE [73], see below), they were excluded from the algorithm summary in Section 3. Briefly, PRIME first decomposes all reversible reactions into forward and backward reactions and iteratively decreases the upper bounds of reactions in the network as much as possible (until biomass production is maintained). It then assesses the correlations between metabolic reaction activities and the measured growth rates. Upper bounds of reactions that are significantly correlated with growth rates are then linearly related to their gene expression values. The pyTARG follows a similar procedure. However, it constraints all the metabolic reactions based on the expression levels of their associated genes [115]. Santos-Beneit et al. applied RNA-seq data from the Human Protein Atlas [23] in combination with pyTARG and HMR metabolic models [117] to reconstruct a context-specific model of healthy lung cells. The metabolic model of SARS-CoV-2 infected cells was obtained with manual curation of the healthy lung cell model using data from the literature that described interactions between viral and human proteins. Moreover, the infected models were augmented with a stoichiometric equation describing the virion production (VBOF). The obtained model was used to identify drug targets by restricting reaction rates catalysed by the tested enzymes. Only the enzymes known to interact with viral proteins were tested and 10 human enzymes were identified as potential targets. Putative inhibitors of selected targets were then assessed with a literature search and by using molecular docking. Based on their analysis, the authors proposed 12 bioactive molecules as promising drugs to treat COVID-19. Some of these are already undergoing clinical trials or have been approved.

Approaches described above mostly relied on a manual adaptation of a selected model to account for the SARS-CoV-2 infection. However, the completeness of a description of a specific context could be increased with the integration of experimental data using context-specific model reconstruction algorithms and protocols (see Section 3 and Section 5). High-throughput data describing the response of different cell lines, tissues, or model organisms were made publicly available shortly after the start of the COVID-19 pandemic (see, e.g., [118]). Several approaches have focused on the integration and analysis of these datasets with genome-scale metabolic models.

Yaneske et al. reconstructed a set of healthy and COVID-19-infected models using transcriptomic and proteomic data from Huh-7 cancer cell lines [74]. Data were obtained from uninfected cells, and cells 24, 48, and 72 h after infection, as reported in [119]. Firstly, the authors expanded the Recon 2.2 model [20] with a VBOF [111] and secretory pathways for a range of relevant immune proteins. Second, data integration and creation of condition-specific models were performed using a modified version of the METRADE pipeline (MEtabolic and TRanscriptomics ADaptation Estimator), in which flux bounds were described by linear functions of differentially expressed gene/protein data and, also, mapping to the phenotype in each condition [73]. They constructed separate models for transcriptomic and proteomic data. Additional constraints were introduced, based on the literature and RNA-seq data analysis results. The analyses of each of the models were performed with FVA evaluating minimal and maximal flux values of each reaction in each of the conditions. These were used to identify differentially active reactions (fold change above the 95th percentile and at least 1.5 for up-regulated reactions, and fold change below the 0.05th percentile and at most 0.8 for down-regulated reactions, respectively). Perturbed metabolic pathways were identified using the hypergeometric test. In their analysis, the authors identified the RNA production, energy production, fatty acid metabolism and the secretome as the main areas of cancer metabolism affected by SARS-CoV-2 infection. [74].

Dillard et al. combined different machine learning approaches with genome-scale metabolic modelling using COVID-19 patient plasma metabolomes (the samples were collected within the proposed study) [104]. The Recon3D model [21] was first adapted to match the measured metabolites with the corresponding metabolites in the model. The exchange bounds of the differential metabolites between the disease states were set to simulate open metabolic exchange. The obtained model was then adapted to non-acute and severe disease states using the RIPTide algorithm [46]. The obtained models were analysed on the basis of 500 FBA samples generated using Gapsplit [120]. Finally, the authors applied random forest classification to identify the reactions capable of differentiating between the non-acute and severe disease models. Using a combination of metabolomic data analysis for biomarker identification and pathways analysis, and genome-scale metabolic modelling for mechanistic understanding, they were able to get a more complete image of COVID-19 impacts on the human body. Furthermore, the top ten identified reactions that accurately classified non-acute and severe disease models agreed with previous research that indicated that interleukin-13 levels drove the severity of COVID-19 disease [121].

Nanda and Ghosh [106] developed healthy and infected models of normal human bronchial epithelial (NHBE) and lung biopsy cells using the data reported in [118]. The models obtained were used to identify metabolic pathways enriched after infection [106]. HumanGEM model [23] was extended with the VBOF before the integration of SARS-CoV-2 infection data using tINIT to obtain the infected models. Healthy models were constructed in a similar way, but without the integration of VBOF and with the integration of data describing uninfected cells. The obtained models were used to generate flux samples. Significantly up- and down-regulated reactions between the healthy and disease states were identified by using the two-sample Kolmogorov–Smirnov test with the Benjamini–Hochberg procedure for false discovery rate control. Finally, only reactions that were altered beyond (for up-regulated) or below (for down-regulated) a predefined threshold were selected as changed. The lists of up- and down-regulated reactions were used to identify enriched metabolic subsystems using the two-tailed hypergeometric test with the Benjamini–Hochberg procedure for false discovery rate control. The authors identified several metabolic pathways that were enriched in the infected NHBE model and could have therapeutic relevance. These included deregulation in fatty acid metabolism, beta-oxidation, and arachidonic acid metabolism, which complied with existing literature data. Furthermore, the authors also identified the reactions affected by post-translational modifications by integrating transcriptomic data with the SARS-Cov-2 protein interaction map [122] and the phospho-proteomic landscape of infection [123], and projecting inhibited enzymes onto metabolic reactions using GPR associations encoded in HumanGEM. The list of affected reactions was again used to perform the metabolic pathway enrichment analysis. The pathways predicted to be most affected were in line with the results from clinical metabolomics studies.

Režen et al. [107] performed an extension of the context-specific GEM analysis described by Nanda and Ghosh [106]. They extended the analysed datasets to also include human embryonic kidney (293T), Calu-3, and adenocarcinoma human alveolar basal epithelial (A549) cell lines [118]. Furthermore, they followed and extended the reconstruction protocol proposed by Walakira et al. [91] to analyse the results obtained with different reconstruction algorithms, including iMAT, INIT, tINIT, and GIMME. GIMME- and tINIT-produced models yielded the most relevant results, allowing a straightforward separation of models by infection and cell type. Their results indicated the modulation of several fatty acid and cholesterol metabolic pathways in COVID-19 patients. The tINIT-produced models also identified the lower metabolism of several vitamins in infected models.

Cheng et al. [102] performed the integration of 12 published gene expression datasets on SARS-CoV-2 infection (3 of which were also analysed in [107]) using the IMAT algorithm, and Recon 1 [19] and Recon3D GEM [21]. Metabolic flux distributions were obtained with flux sampling and differential fluxes were assessed between the healthy and infected models. Reactions with altered fluxes were identified by observing the absolute rank biserial correlations and absolute relative flux changes (>0 for up- and <0 for down-regulated reactions, respectively). The enriched reaction sets were compared across datasets using Fisher’s exact test. Since no reaction was common to all datasets, the most relevant reactions were identified by the intersection between bulk RNA-seq patient datasets and single-cell RNA-seq (scRNA-seq) datasets. These reactions were then used to perform pathway enrichment analyses using Fisher’s exact test. The latter was performed separately on the Recon 1 and Recon3D models, and pathways with inconsistent enrichment results were removed from further analysis. The authors visualised selected enriched pathways in which directions of edges (reactions) were determined by the consensus direction and differential fluxes through reaction with the consensus across the datasets. Furthermore, robust metabolic transformation analysis (rMTA) [124] in flux samples was used to predict single targets or targets in combination with remdesivir (RNA-seq data from Vero E6 cells infected by SARS-CoV-2, with or without remdesivir treatment), whose inhibition facilitates the transformation from diseased to healthy states. The predictions obtained were validated on different datasets with anti-SARS-CoV-2 gene targets or drugs. Furthermore, the top 10 % of the rMTA targets were used to obtain a set of consensus candidate targets in the data sets, which were assigned to known drugs using the DrugBank database [113]. Additionally, the authors performed the metabolic pathway enrichment analysis of identified targets (Fisher’s exact tests) to prioritise the ones for experimental validation, using siRNA assay in Caco-2 cells. The results obtained with pathway-based analyses complied with previous studies, which indicated that GEMs could be used for antiviral target prediction. This was confirmed with the validation of the identified drug targets using literature data and additional validation experiments.

Kishk et al. proposed another pipeline for GEM-based drug repurposing using FBA [103]. As in most other works, the generic models (Recon 2 [20] and Recon3D [21]) were extended with a VBOF according to [98]. Context-specific models were obtained using two RNA-seq studies on healthy, mock and infected lung expression data with different disease severity levels [118,125]. The blocked reactions were removed using FASTCC [40] and the integration of data was performed using rFASTCORMICS [42]. Potential drug targets were identified with single (essential genes) and double (synergetic genes) gene knockouts using the COBRA toolbox [9]. Double knockouts revealed two types of gene-pair combinations, namely, pairs of non-essential genes that reduced the viral growth when silenced simultaneously, and pairs of essential and non-essential genes that induced a stronger reduction when silenced simultaneously. The safety of essential genes for healthy tissue was assessed with their impact on biomass in different healthy models. Essential and synergistic genes were used to perform KEGG pathway enrichment analysis and GEM-based metabolic pathway enrichment analysis. Existing drugs targeting the predicted essential genes were identified in the DrugBank database [113]. Drugs with multi-target effects were identified by constructing the drug–gene–pathway interaction networks. A set of 85 repositionable single drugs, 47 drugs on gene pairs, and 52 drug combinations against COVID-19, were identified.

Ambikan et al. presented multi-omics (blood RNA-seq and plasma metabolomics) analyses of personalised networks in which patients were stratified according to the severity of the disease, and which also included reconstruction and analysis of personalised and group-specific GEMs [108]. Human-GEM [23] was used as a reference model in the reconstruction. Personalised models were constructed with individual gene expression data and group-specific models were constructed with average gene expression data, using the tINIT algorithm. Exchange reactions were constrained on the basis of metabolomics data and validated with the literature data [126]. VBOF was incorporated into the models built with COVID-19-positive samples. For infected models, FBA was used to maximise the flux through a pseudo-reaction with VBOF products and ATP hydrolysis as reactants. In other models, ATP hydrolysis was used as an FBA objective function. Based on group-specific GEMs, the authors identified 100 reactions that were differentially active between patient groups and 15 transport reactions that were differentially active when comparing mild/moderate and severe groups with healthy controls. Personalised GEMs were used to identify 274 differentially active reactions among patients, mostly consistent with the results of the group-specific analysis. The consensus among the models yielded 16 specific reactions to COVID-19 and 10 specific reactions to COVID-19 severity. The authors additionally performed a network topology analysis on the set of metabolites and enzymes present in active (non-zero flux) reactions. This analysis was used to identify network communities and to prioritise nodes (metabolites and enzymes) based on their centrality values. Seven genes and 51 metabolites were identified as prioritised nodes. Single-gene deletion COBRA function [9] was used to identify essential genes in COVID-19 patients, these being the mitochondrial genes. This was in concordance with other analyses in the study which exposed the central metabolic pathway, and the TCA (tricarboxylic acid) cycle, as essential in COVID-19 patients.

Renz et al. described a computational pipeline for the identification of broad-spectrum antiviral drugs using context-specific GEMs [109]. The authors extended the Recon 2.2 [20] with the VBOF as described earlier. The generic model was then adapted using FASTCORE [40] and StanDep scRNA-seq preprocessed data [89]. Viral replication capacities across different cell types were assessed using the FBA on the models reconstructed with the integration of mice data from the Tabula Muris Consortium dataset [127] and data from humans covering the gastrointestinal tract [128]. In accordance with the past observations, the highest viral replication capacities were observed for the intestine and cells of the oral cavity [109]. Viral replication capacities were additionally investigated in COVID-19-specific patient GEMs that were obtained with the integration of scRNA-Seq data from COVID-19 patients [129]. In these models, viral replication capacity was strongly increased in the upper respiratory tract, and ciliated, secretory and FOXN4 cells showed a mean increase in comparison to uninfected models. Metabolic pathway analysis revealed 55 enriched pathways (out of 57 analysed pathways) in severely diseased patients, and 39 enriched pathways in moderately and severely diseased patients, in comparison to healthy controls. Furthermore, the authors used single gene deletions to predict different types of antiviral targets, where targets which decreased the VBOF by at least 50 % and which did not strongly affect the biomass of a cell (did not fall below 80 % of the initial value) were identified as primary targets. Among these, potential broad-spectrum antiviral targets that occurred across all cells from the individual datasets were selected. Moreover, only the targets that had already been reported to interact with other human pathogenic viruses were used to finally identify four enzyme targets. These were applied in further experimental validation using SARS-CoV-2 infected Calu-3 and CaCo-2 cell lines. The latter confirmed Phenformin and Atpenin A5 as potential broad-spectrum antiviral drugs.

Finally, Thiele and Fleming generated a whole-body metabolic sex-specific host-virus model (WBM) [110] based on previously reported WBMs of human metabolism [126]. The latter was extended with a VBOF and with other SARS-CoV-2 specific reactions formulated using available knowledge, including virus uptake through the air, replication in different tissues, degradation by CD4+ T cells, and release back into the air. Organs and cells that could be affected by the virus included the lung, CD4+ T cells, adipocytes, small intestinal epithelial cells, and liver. In total 25 virus-specific reactions were included in the WBMs. Furthermore, the models obtained were additionally constrained according to the physiological and dietary parameters of individuals. The authors assessed the metabolic flux distributions using FBA that minimises the Euclidian norm (to obtain a unique solution) and with the flux through a virus shedding reaction as an objective. The models were able to yield a feasible solution with the basic viral load (modelled with the flux through the viral uptake reaction). However, an increase in T cells was required to obtain a feasible solution in models with the amount of viral load corresponding to hospitalised and severe COVID-19 patients, which was consistent with current knowledge. Furthermore, the authors assessed metabolic changes associated with the infection, disease severity (viral load), and CD4+ T cell availability in three models for each sex. When comparing healthy models with infected models, and comparing both infected models, the flux values changed by at least 10 % in approximately 15 % of reactions, demonstrating that the metabolism of the entire body is affected during the viral infection. The authors additionally used the established models to analyse the blood metabolome by calculating the maximally possible increase or decrease of metabolites in the blood compartment with the addition and individual maximisation of artificial reactions that allowed the accumulation of each metabolite. The change of the metabolome showed good agreement with the literature and was observed in around 35 % of the metabolites between healthy and infected models and between infected models with different disease severity levels. Furthermore, the authors used their models to analyse different sets of drug targets previously reported in the literature. Although the models were unable to confirm the results reported in [122], they produced results consistent with a previous study on GEMs [98]. Since in silico analyses indicated that isoleucine was a rate limiting factor for the viral shedding rate, the authors also inhibited different lung amino acid uptake (isoleucine, threonine, tryptophan, and lysine), resulting in a reduction in shedding rate. These perturbations could also be achieved by dietary changes. The authors additionally analysed the effects of different diets on viral shedding flux. The Virtual Metabolic Human database [130] was used to establish different diets and the lowest shedding rate was achieved with the vegan and vegetarian diets. On the other hand, burger- and steak-rich diets yielded the highest virus replication rates. Finally, the authors used the WBM models to analyse viral shedding and replication rates, and amino acid requirements of different virus variants described with different VBOFs.

## 7. Conclusions

Even though several standard bioinformatic approaches for the analyses of omics data have been applied in the context of COVID-19 applications (e.g., see [131]), GEM-based analyses complement these approaches, since they are able to provide additional insights into experimental data. The main benefits of GEM-based applications include a mechanistic view into the dynamics of the disease, as well as possibilities to test different hypotheses in silico (e.g., using in silico gene knockouts).

Our understanding of COVID-19 has been greatly enhanced with the employment of GEM-based analyses. These analyses have been conducted on context-specific models reconstructed with a combination of manual curation and algorithms for automated reconstruction, as reviewed in this paper. The latter have employed different types of experimental data, such as data obtained from different cell lines (e.g., see [74]), patient samples (e.g., see [108]), or a combinations of both (e.g., [102]). Most of the approaches devoted to the GEM-based analysis of COVID-19 focused on the identification of enriched metabolic reactions and pathways (e.g., see [102,106,107]) and/or to the identification of potential antiviral targets (e.g., see [98,99,105]) and drug repurposing (e.g., see [100,103]). Reported studies also focused on the identification of metabolic reactions guiding disease severity [108], and on the analysis of cancer metabolism affected by SARS-CoV-2 infection [74]. The approaches analysed different SARS-CoV-2 variants (e.g., see [99,105]) and infection dynamics in different cell types [109]. Finally, whole-body models were applied to analyse the consequences of the infection on the whole human body [110]. Context-specific GEMs have, thus, been able to confirm existing COVID-19 treatment strategies, as well as resulted in proposals of novel drug targets and repurposed drugs for effective treatment. However, validation of the obtained results with additional experiments has so far been limited. Moreover, most of the approaches were limited to the analysis of the metabolism of specific cell types in isolation.

It is a fact that automated approaches have allowed for fast and straightforward reconstruction of context-specific models. However, even though we can, at least to some extent, rely on such automation, the quality of reconstructed models is still strictly dependent on the quantity and quality of biological knowledge incorporated into a generic model of an organism. For example, most of the reconstruction approaches rely on the associations between genes and metabolic reactions defined by GPR rules encoded within the reference model. If this data is inaccurate or is missing relevant associations, the reconstruction process cannot yield a biologically relevant model.

Specific GEM repositories, such as BiGG [132] and Metabolic Atlas [23] have already been reported on. However, these repositories are mainly focused on publishing generic GEMs. Having (parts of these) repositories specifically devoted to context-specific GEMs, which would be searchable through parameters, such as an algorithm used for the reconstruction or focus of the reconstruction (e.g., disease, tissue, etc.), would ease the reproducibility and reusability of the reconstructed context-specific GEMs.

Another aspect that should also be addressed in the context of reproducible models with large biological significance is the establishment of standards, protocols and tools for automated reconstruction of context-specific GEMs. Even though certain attempts have already been made in this direction (see Section 5) an easy-to-use tool that would automatically select the best reference model and algorithm(s) for a specific problem (i.e., dataset), perform parametrisation of these algorithms, validate the obtained results and yield a set of the most significant models has not yet been reported.

Another limiting factor of GEMs in general is that they describe the dynamics of a metabolic network in isolation. The majority of GEMs have been focused on a model of an individual cell, and are, thus, unable to capture the metabolism of the whole body. Recently, Thiele et al. presented a set of personalised sex-specific whole-body metabolic (WBM) GEMs [126]. These were also applied in the context of the analysis of metabolic reprogramming followed by SARS-CoV-2 infection [110]. Even though WMB models could describe the metabolism of a large number of organs in the human body [110], they only account for the (steady-state) response of metabolic networks, which are isolated from other biological networks. Several integrative approaches to connect metabolism to other cellular processes have already been proposed. For example, much effort has been devoted to the integration of GEMs with gene regulatory networks [133] and other regulatory mechanisms [134]. Moreover, GEMs have been integrated into whole-cell models, but only for simple organisms, such as *Mycoplasma genitalium* [135], and more recently for *Saccharomyces cerevisiae* [136]. However, the integration of such models into a human whole-cell model [137] and the virtual human body [138] is still less developed [126].

Despite these facts, GEMs have proven to be an efficient way to incorporate high-throughput data into mathematical representations. These can not only be used to perform computational simulations and assess metabolic reaction activities, but can also comprehensively capture knowledge about the metabolic functions of a cell [139]. Moreover, despite several presumptions and simplifications applied within the GEM reconstruction and analysis, GEMs are able to provide valuable insights into the metabolism of a specific organism and its context-specific reprogramming, as also demonstrated by the COVID-19 applications described in this paper.

## Figures and Tables

**Table 1:** An overview of different families of algorithms for context-specific model reconstruction. Abbreviations: RMF—required metabolic function; MILP–mixed integer linear programming.

Family	Description
GIMME-like	Maximising the compliance with the experimental evidence while pertaining to a given RMF.
iMAT-like	Does not specify a RMF, matching of reactions states (active or inactive) with expression profiles (present or absent), employs MILP-based optimisation.
MBA-like	Defining core reactions and removing other reactions while pertaining to model consistency, support integration of different data types.
MADE-like	Employs differential gene expression data to identify flux differences between two or more conditions.

**Table 2:** An overview of algorithms for automated reconstruction of context-specific models. Abbreviations: LP—linear programming; RMF—required metabolic function.

Algorithm	Reference	Family	Input Data	Comments
GIMME	Becker et al., 2008 [38]	GIMME-like	transcriptomics	Inactivate reactions below a threshold while maintaining RMF.
GIMMEp	Bordbar et al., 2012 [44]	GIMME-like	transcriptomics, proteomics	RMFs based on proteomics data.
GIM3E	Schmidt et al., 2013 [45]	GIMME-like	transcriptomics, metabolomics	No thresholding.
RIPTiDe	Jenior et al., 2020 [46]	GIMME-like	transcriptomics	Minimises the weighted flux values, no thresholding.
iMAT	Zur et al., 2010 [47]	iMAT-like	transcriptomics, proteomics	Matches reaction activities with expression profiles, no RMF.
INIT	Agren et al., 2012 [48]	iMAT-like	transcriptomics, proteomics, metabolomics (qualitative)	Reaction weights based on experimental evidence, integration of metabolomics data.
tINIT	Agren et al., 2014 [49]	iMAT-like	prior knowledge, transcriptomics, proteomics, metabolomics (qualitative)	Based on a set of required metabolic tasks.
Lee	Lee et al., 2012 [50]	iMAT-like	transcriptomics	Uses absolute expression data (RNA-seq).
RegrEx	Estevez et al., 2015 [51]	iMAT-like	transcriptomics	Uses absolute expression data (RNA-seq) and regularisation.
MBA	Jerby et al., 2010 [52]	MBA-like	prior knowledge, transcriptomics, proteomics, metabolomics, fluxomics	Removes non-core reactions and checks model consistency for core reactions.
mCADRE	Wang et al., 2012 [53]	MBA-like	transcriptomics, metabolomics	Different reaction scores to determine core reactions.
FASTCORE	Vlassis et al., 2014 [40]	MBA-like	a set of core reactions	Two LPs to find a minimal set of non-core reactions to activate all core reactions.
SWIFTCORE	Tefagh and Boyd, 2020 [54]	MBA-like	a set of core reactions	Enhanced runtime and network compactness in comparison to FASTCORE.
FASTCORMICS	Pires Pacheco at al., 2015 [41]	MBA-like	transcriptomics	FASTCORE workflow for microarray data.
rFASTCORMICS	Pires Pacheco at al., 2019 [42]	MBA-like	transcriptomics	FASTCORE workflow for RNA-seq data.
scFASTCORMICS	Pires Pacheco at al., 2022 [55]	MBA-like	transcriptomics	FASTCORE workflow for scRNA-seq data.
CORDA	Schultz and Qutub, 2016 [34]	MBA-like	a set of core reactions	Does not require to remove all non-core reactions.
MADE	Jensen and Papin, 2011 [56]	MADE-like	transcriptomics	Identifies reaction activities in a sequence of measurements.
RMetD2	Zhang et al., 2019 [57]	MADE-like	transcriptomics	Sequentially pushes the constraints.
ΔFBA	Ravi et al., 2021 [58]	MADE-like	transcriptomics	Finds a consistent and minimal solution of flux differences between the conditions.

**Table 3:** The types of high-throughput data, their use and available repositories.

High-Throughput Data	Input Data	Algorithm	Data Repositories
Transcriptome	Gene expression value	GIMME-like	ArrayExpress
		iMAT-like	cBioPortal
		MBA-like	CCLE
		PRIME	EGA
	Differential gene expression value		ENA
			Expression Atlas
			FANTOM5
		MADE-like	GEO
		METRADE	GTEx
			HPA
			SRA
			TCGA
Proteome	Protein expression value	GIMME-like	cBioPortal
		iMAT-like	CCLE
		MBA-like	Expression Atlas
	Differential protein expression value		HPA
			PDC
		METRADE	ProteomeXchange
			TCGA
Metabolome	Metabolite concentration	GIMME-like	MetaboLights
		iMAT-like	Metabolomics workbench
		MBA-like	

**Table 4:** An overview of applications of genome-scale metabolic modelling and analysis of COVID-19. Abbreviations: VBOF—viral biomass objective function, WBM—whole-body model.

Reference	Reconstruction Algorithm(s)	Comments
Renz et al., 2020 [98]	none	Integration of VBOF into a human alveolar macrophage model.
Renz et al., 2021 [99]	none	A follow-up study on [98].
Delatre et al., 2021 [100]	none	Integration of VBOF into a human lung cell model.
Yaneske et al., 2021 [74]	METRADE	A combination of manual curation with automated reconstruction using transcriptomics and proteomics data from Huh-7 cells.
Santos-Beneit et al., 2021 [101]	pyTARG (for a healthy lung model)	Manual curation of a healthy lung model with literature data.
Cheng et al., 2021 [102]	iMAT	An integration of data from 12 datasets, validation of identified targets with additional experiment.
Kishk et al., 2021 [103]	rFASTCORMICS	An integration of data from two RNA-seq studies on lung cells.
Dillard et al., 2022 [104]	RIPTide	Combining GEMs with machine learning analysis on plasma metabolomes of non-acute and severe COVID-19 patients.
Wang et al., 2022 [105]	none	Extension and integration of VBOF into Recon3D.
Nanda and Ghosh, 2021 [106]	tINIT	An integration of NHBE and lung biopsy RNA-seq data into HumanGEM.
Režen et al., 2022 [107]	GIMME, iMAT, INIT, tINIT	An integration of different cell lines and patient samples data following the protocol proposed in [91].
Ambikan et al., 2022 [108]	tINIT	A reconstruction of personalised and group-specific models with integration of RNA-seq data (blood), constraining exchange reactions with metabolomics data (plasma).
Renz et al., 2022 [109]	FASTCORE	A computational pipeline for identification of broad-spectrum antiviral drugs using scRNA-seq data.
Thiele and Fleming, 2022 [110]	none	An integration of VBOF and other virus-specific reactions into metabolic sex-specific WBM.

## Abbreviations

The following abbreviations are used in this manuscript:

ATP	adenosine triphosphate
BOF	biomass objective function
CCLE	Cancer Cell Line Encyclopedia
COBRA	constraint-based reconstruction and analysis
CORDA	cost optimization reaction dependency assessment
COVID	coronavirus disease
EBI	European Bioinformatics Institute
EGA	European Genome-Phenome Archive
ENA	European Nucleotide Archive
FANTOM5	Functional Annotation of the Mammalian Genome 5
FASTCC	fast consistency checking
FBA	flux balance analysis
FVA	flux variability analysis
GEM	genome-scale metabolic model
GEO	Genome Expression Omnibus
GIM3E	gene inactivation moderated by metabolism, metabolomics and expression
GIMME	gene inactivity moderated by metabolism and expression
GIMMEp	gene inactivity moderated by metabolism and expression by proteome
GPR	gene-protein-reaction
GTEx	Genotype-Tissue Expression database
HPA	Human Protein Atlas
iMAT	integrative metabolic analysis tool
INIT	integrative network inference for tissues
LP	linear programming
MADE	metabolic adjustment by differential expression
MBA	model building algorithm
mCADRE	metabolic context-specificity assessed by deterministic reaction evaluation
METRADE	MEtabolic and TRanscriptomics ADaptation Estimator
MILP	mixed integer linear programming
MIQP	mixed integer quadratic programming
MTA	metabolic transformation algorithm
NCBI	National Center for Biotechnology Information
NGS	next-generation sequencing
NHBE	normal human bronchial epithelial
PCA	principal component analysis
PDC	Proteomic Data Commons
pFBA	parsimonious flux balance analysis
PRIME	personalized reconstructIon of metabolic models
QP	quadratic programming
RegrEx	regularized context-specific model extraction method
RIPTide	reaction inclusion by parsimony and transcript distribution
RMetD2	relative metabolic differences version 2
RMF	required metabolic function
RNA-seq	RNA sequencing
SARS-CoV-2	Severe acute respiratory syndrome coronavirus 2
scRNA-seq	single cell RNA sequencing
SRA	Sequence Read Archive
TCGA	The Cancer Genome Atlas
TIGER	toolbox for integrating genome-scale metabolism, expression, and regulation
tINIT	task-driven integrative network inference for tissues
TPM	transcripts per million
VBOF	viral biomass objective function
WBM	whole-body model
